# A review of structural and functional brain networks: small world and atlas

**DOI:** 10.1007/s40708-015-0009-z

**Published:** 2015-02-14

**Authors:** Zhijun Yao, Bin Hu, Yuanwei Xie, Philip Moore, Jiaxiang Zheng

**Affiliations:** School of Information Science and Engineering, Lanzhou University, Lanzhou, China

**Keywords:** Brain networks, Atlas, Small world

## Abstract

Brain networks can be divided into two categories: structural and functional networks. Many studies of neuroscience have reported that the complex brain networks are characterized by small-world or scale-free properties. The identification of nodes is the key factor in studying the properties of networks on the macro-, micro- or mesoscale in both structural and functional networks. In the study of brain networks, nodes are always determined by atlases. Therefore, the selection of atlases is critical, and appropriate atlases are helpful to combine the analyses of structural and functional networks. Currently, some problems still exist in the establishment or usage of atlases, which are often caused by the segmentation or the parcellation of the brain. We suggest that quantification of brain networks might be affected by the selection of atlases to a large extent. In the process of building atlases, the influences of single subjects and groups should be balanced. In this article, we focused on the effects of atlases on the analysis of brain networks and the improved divisions based on the tractography or connectivity in the parcellation of atlases.

## Introduction

Studies dating from the nineteenth century have demonstrated that neuronal elements construct an extremely complicated structural network [1]. Currently, studies of the topological structure of brain networks and the relationship between structure and brain function remain a tremendous challenge [2]. Although knowledge of the neuroscience of molecular and genetic mechanisms has increased, the principles of cognition are not generally understood. Therefore, the relationship between consciousness and higher brain functions requires further investigation [3]. There is a clear need for new methods to study the brain, which is a complex and generally misunderstood dynamic system.

Modern theories of networks, originating from mathematics and sociology, are valuable methods used in the study of the complex systems [4]. The brain network is the complex network that supports efficient information integration and communication with relatively low wiring costs [5]. Recently, some network models have been applied to demystify the structural characteristics of brain networks and the basement of brain functional networks, such as the small-world network [6] and scale-free network [7, 8]. The small-world network, characterized by a high clustering coefficient and short path length, was described by Watts and Strogatz [6]. Many previous studies have demonstrated that structural and functional brain networks are characterized by a small-world architecture [9–14]. The scale-free network is characterized by an average small number of connections of each node, but with a high level of global connectivity guaranteed by the existence of a small number of highly connected nodes [7, 8, 15]. However, another study reported that functional networks based on resting-state functional MRI (fMRI) data at the macroscale followed an exponentially truncated power law distribution [16]. The analysis results of anatomical networks in humans also showed a degree of distribution following the truncated power law [17].

**Fig. 1:**
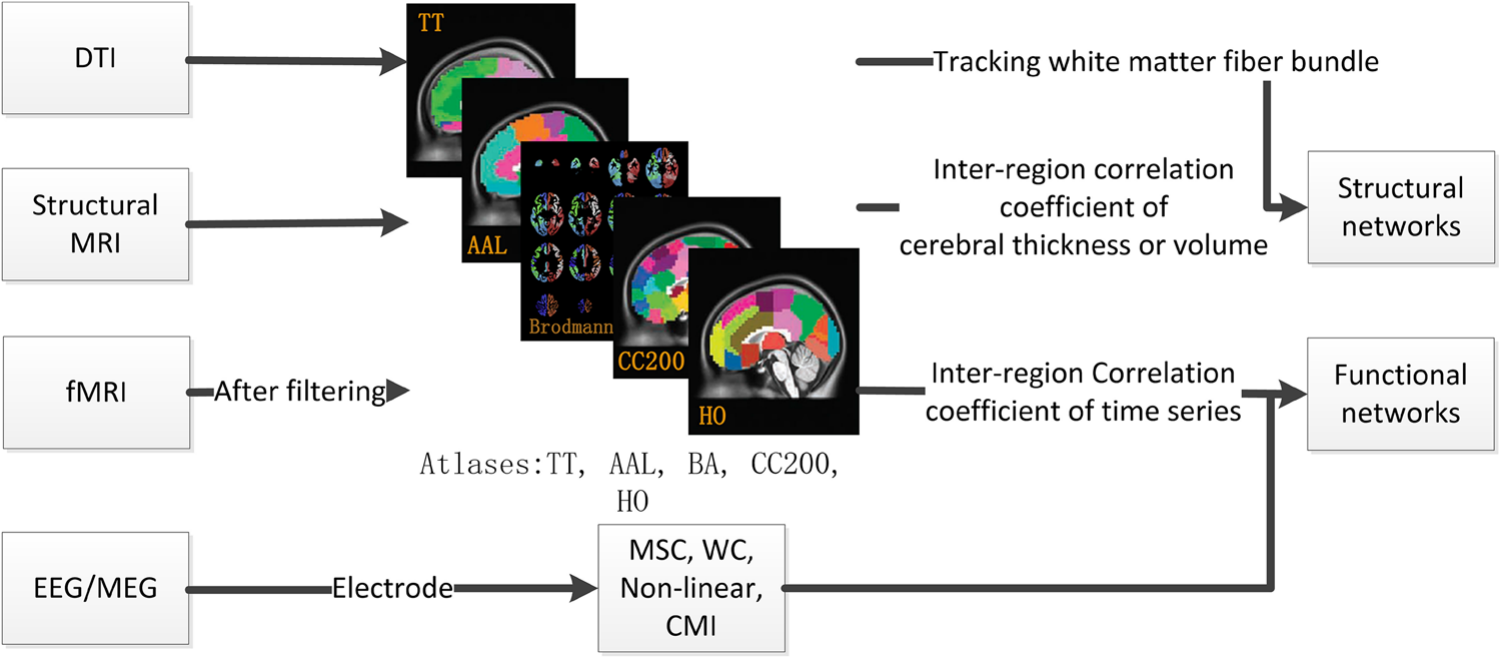
The construction of functional and structural networks

In the analysis of structural and functional networks, some predefined parcellation atlases, the automated anatomical labeling (AAL) and automatic nonlinear imaging matching and anatomical labeling (ANIMAL) atlases, have been widely used [12, 16, 18, 19, 20]. In addition, still some other atlases were used in the previous studies, such as the Harvard-Oxford (HO) atlas derived from anatomical landmarks (sulci and gyral), the Eickhoff-Zilles (EZ) atlas derived from cytoarchitectonic segmentations, the Talariach Daemon (TT) atlas derived from myeloarchitectonic segmentations and the LONI Probabilistic Brain Atlas (LPBA40) derived from population-based probability [21, 22]. More information on the above atlases can be obtained from [23]. Since the importance of determining the nodes in brain networks was proposed, researchers have made many efforts toward atlas optimization. Several previous studies have reported the impacts of different atlases on brain networks [24, 25]. Quantification of specific brain network attribute parameters was obviously affected by atlases, such as the clustering coefficient and shortest path length. Actually, quantification of the brain network was directly affected by parcellation strategies, such as connection weights, which limit the selection of network models and parameters. Also in the previous studies, the credibility results were archieved by the analysis of simplified networks, such as binary networks. In this article, we discuss recent studies on parcellation of the brain and the impact of atlases on the construction of brain networks.

## Brain networks and atlases

### Structural and functional networks

In graph theory, the network is a graph consisting of a set of nodes with connecting edges. The brain network can be defined by a connection matrix (a graph theory concept), also called a connectome [26]. The node in the connection matrix is the key element, and it is still an unclear concept in the analysis of brain networks [26]. Scale and parcellation are key restricted factors in the definition of nodes. The number of the edges connecting a node is called the degree. The edges of brain networks can be weighted or unweighted, directed or undirected. Structural and functional networks can be processed as the simplest graph (an unweighted and undirected graph). However, weighted networks and directed networks cannot be ignored despite the existing controversy. The construction of brain networks is showed in Fig. 1 and described below.

The methods to construct structural brain networks can be divided into two categories: cerebral cortex correlation and white fiber tracking. Many types of brain morphological measurements are used to calculate cerebral cortex correlation, including the commonly used cortical thickness and volume [27–29]. Anatomical networks can be obtained by calculating correlations of cortical thickness (or volume) between all pairs of regions in a predetermined anatomical parcellation scheme, such as in the AAL and ANIMAL atlases [12, 30]. Strong interregional correlation of cortical thickness measurements may be the axonal connection, which might be caused by mutual nutrition [31–33]. Structural networks can also be constructed by the data of diffusion tensor imaging (DTI) via tracking the white matter fiber bundles [34]. Some summation indices, such as the trace apparent diffusion coefficient or the fractional anisotropy (FA), can be extracted using tensor decomposition from DTI [35–37]. DTI has become the preferred choice for detecting white matter alterations in the human brain [38]. However, the partial volume effect and inability of the model to cope with nonGaussian diffusion are the two main drawbacks of DTI [39], which limit its application.

Functional brain networks consist of separated brain regions and functional connectivities between pairs of brain regions. Functional connectivity is defined as the temporal dependency of neuronal activation patterns of anatomically separated brain regions [40]. The low-frequency oscillations (~0.01–0.1 Hz) of blood oxygenation level-dependent fMRI time series recorded during the resting state are gaining special attention and are used to show correlated patterns between separate brain regions [41, 42]. Although many methods can be used to measure spontaneous brain activities and the correlation between these activities and some neurological diseases in resting-state fMRI, the test-retest reliability of connectivities is still unclear [43]. Specifically, a small intraindividual variability and large interindividual variability can lead to high test-retest reliabilities [43]. Most previous studies of functional networks were based on atlases divided by anatomical or cytoarchitectonic boundaries [44, 45]. As the reliability and suitability of these approaches were unclear, building atlases based on functional connectivity was imperative. Recently, some functional atlases have been proposed, such as the Dosenbach 160-region atlas and Power 264-region atlas, both generated based on metaanalysis of task-related fMRI data [46, 47].

Compared with fMRI, EEG and MEG have a higher frequency and wider band (~1 to 100 Hz) but lower spatial resolution. Because of the high temporal resolution, EEG and MEG are useful techniques in the study of brain dynamics and functional connectivity [48]. The functional connectivity could be measured via linear methods, such as cross-correlation of pairs of EEG signals [49], magnitude-squared coherence and wavelet coherence from EEG [50]. Besides linear methods, nonlinear methods on the basis of deterministic chaos and information-based techniques can also be used to measure the functional connectivity in EEG/MEG. Cross mutual information is a typical information-based method that has been used to diagnose Alzheimer’s disease and schizophrenia [51–53].

### Atlases in structural and functional networks

Atlases can be considered as a bridge between neuroimaging data and graph theory analysis on the macroscale. Neuroimaging data can be converted into graph theory elements (such as nodes) through atlases. Adverse effects of using atlases in network analysis should be reduced as much as possible because individual variables (such as head motion) can affect subsequent network analysis after parcellation. Quantification of brain networks will be affected by these individual variables. These mentioned impacts are present in the widely used atlases, such as the AAL, ANIMAL and Brodmann [12, 16, 18, 19, 20, 54, 55]. Previous studies have shown that small-world properties in brain networks are robust, morphology independent and atlas independent. Small-world properties are determined by γ and λ, and they can be worked out as follows: $\gamma = C^{real}_p/C^{random}_p>1 {\rm and}\, \lambda = L^{real}_p/L^{random}_p\approx 1\, {\rm where}\, C^{random}_p\,{\rm and}\,\,L^{random}_p$ are the mean network clustering coefficient and the mean network absolute shortest path length of matched random networks that have the same number of nodes, edges and degrees distributions [6]. He et al. detected the small-world properties of brain networks from the cerebral cortex thickness divided by the ANIMAL atlas [12]. Small-world properties of brain network were also studied based on the brain volume divided by the AAL atlas [56]. In these analysis courses of the brain structure network, some different parameters and steps were used, and the key point was the use of different atlases. These differences might make a major obstacle to the comparison between these results in structural networks. In functional networks, nodes were widely identified based on the Brodmann areas [57–59]. At the same time, small-world properties in functional networks were studied based on the AAL atlas [16, 60]. Of course, some functional atlases were used to study small-world properties from fMRI [24, 25, 55, 61].

**Fig. 2:**
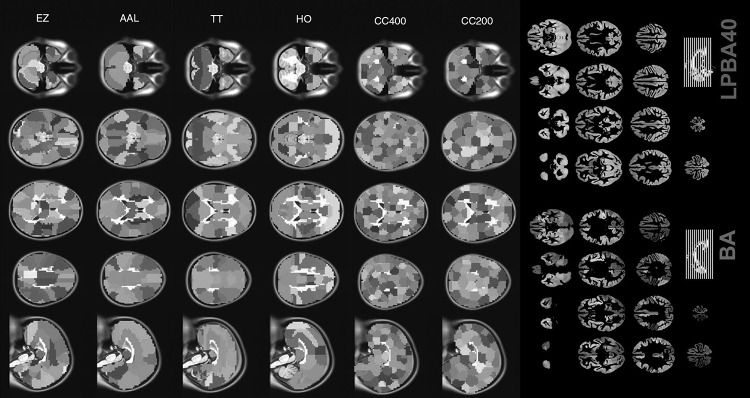
This figure shows information on the * AAL*,* EZ*,* TT*,* HO*,* CC200*,* CC400*,* Brodmann (BA)* and* LPBA40* atlases [21, 62]

The same atlas provided convenient comparison of the structural and functional networks, but the differences in small-world properties between functional networks constructed by the ANIMAL and AAL atlases respectively have been a concern [24]. Besides the small-world properties, the degree distribution was also affected [24]. Wang et al. found that small worldness and the degree distribution were significantly different in brain networks based on different atlases [24]. However, different atlas parcellation strategies did not affect whether a small-world structure or an exponentially truncated power law distribution existed. These differences might be caused by the region size or method used to obtain the atlas, which indicates the importance of selection in atlases.

Zalesky et al. studied the properties of anatomical networks on the macroscale (like the AAL and ANIMAL atlases) and mesoscale. They reported a similar result, finding that the node scale did not affect whether or not a network was small world or scale free, but the scale affected the quantification and the extent to which the network exhibited these topological properties [25]. Andrew et al. constructed an anatomical network using DTI data and reported that small worldness exhibited a 95 % difference between the widely used AAL template and a 4,000-node random parcellation ($\sigma _{AAL}=1.9\,{\rm\,vs.}\,\sigma _{4000}=53.6\pm 2.2$ [25]. More nodes with higher spatial resolution can preserve more individual differences in fMRI analysis. Therefore, the combination of different node scales was necessary [61].

As discussed above, different atlases can be applied for different neuroimaging data to find some similar network properties, but discrepancies in the properties exist, which means that atlases are an important factor in the reliability of brain network analysis. Although the AAL and Brodmann atlases were popular, they might perform more poorly than other atlases, such as the LPBA40 [22]. Ota et al. showed that the LPBA40 performed better than the Brodmann and AAL atlases in predicting mild cognitive impairment [62]. Details of the Brodmann, AAL and LPBA40 atlases are shown in Fig. 2.

## Improvement in parcellation

**Fig. 3:**
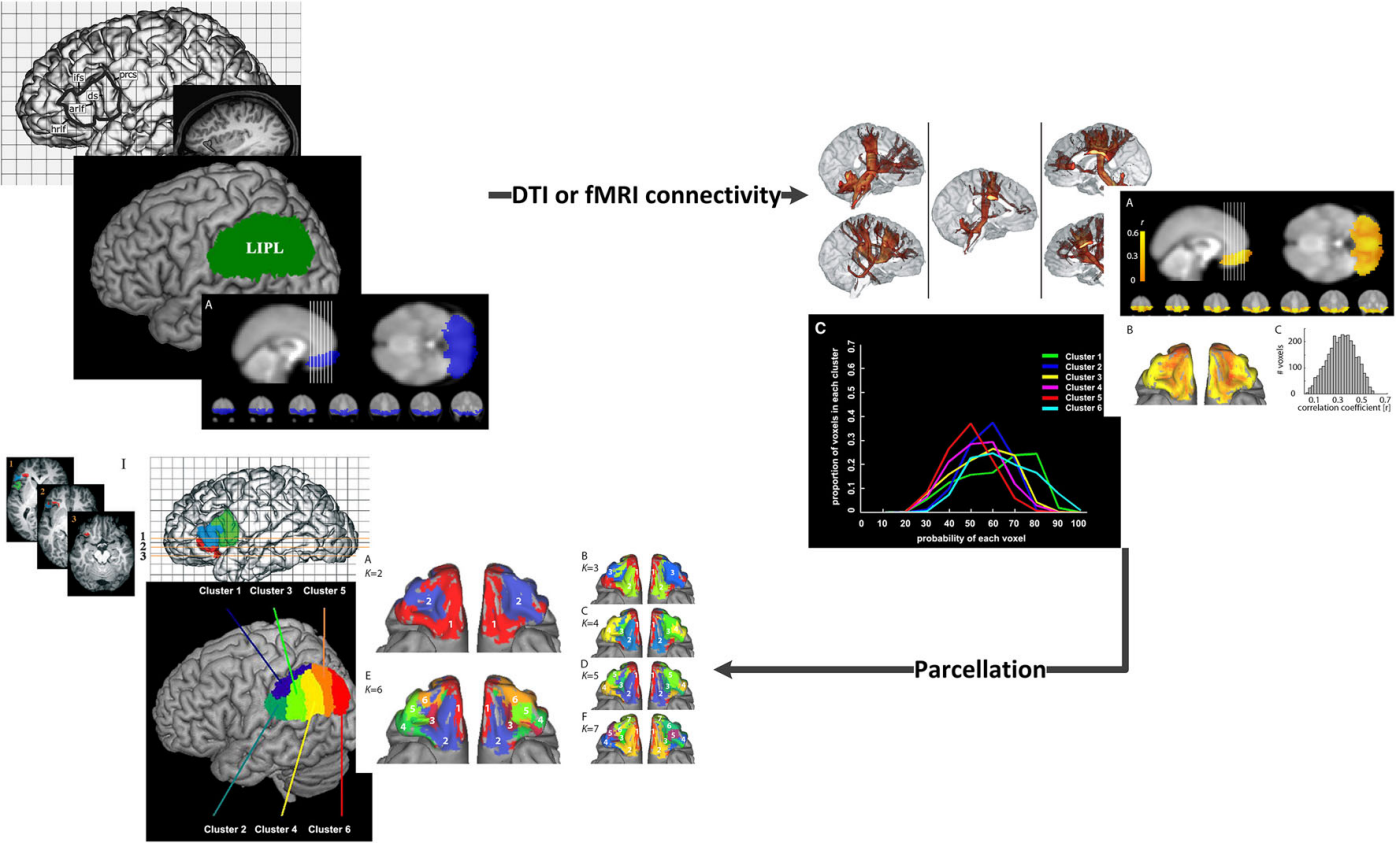
The parcellation process based on DTI and functional connectivity. This figure shows parcellations of the substantia nigra [64], Broca’s area [65] and left inferior parietal lobule [66] based on DTI and parcellations of the human orbitofrontal cortex based on resting-state fMRI [71]

Research has been carried out addressing brain networks, comparing functional (resting-state fMRI) and structural networks (diffusion-based methods). The previous studies suggested that diffusion-imaging and fMRI data reveal a close relationship between structural and functional connections, including some brain regions that form the structural core [63]. The combination of DTI and fMRI can improve cortex parcellation. The internal diversity of some regions with heterogeneous functions and anatomy could be subdivided by DTI, such as Broca’s area, the supplementary motor area (SMA), posteromedial cortex (PMC), substantia nigra and left inferior parietal lobule (LIPL) (see Fig. 3) [64–68]. The somatotopic representation of the body and temporal organization of movements were partially controlled by the SMA [69]. The PMC had the following functions: visuospatial imagery, episodic memory retrieval, self-processing and consciousness [70]. Of course, cortex parcellations can also be identified based on a* K*-means cluster algorithm using only fMRI data [71]. These parcellations depending on DTI or fMRI data were mainly based on unsupervised clustering techniques, such as the K-means cluster and spectral cluster algorithms [72, 73]. Recently, some atlases based on clusters of functional connectivity were proposed. Craddock et al. reported a functional atlas (the CC200 in Fig. 2) generated based on spatially constrained spectral clustering [74].

Although clustering-based parcellations could improve the divisions of some heterogeneous regions to some extent, some disadvantages still needed more study, such as the reproducibility and hierarchy. The atlases used could be divided into single-subject topological and population-based probabilistic atlases [75], single-subject atlases revealing more interindividual differences and population-based probabilistic atlases revealing more intergroup differences. The parcellation should be balanced between interindividual and intergroup differences, revealing more disease-related differences and the individual effects on them.

Small-world properties can be destroyed by neuropsychiatric diseases. Liu et al. reported that topological measurements such as clustering and small worldness were inversely correlated with the duration of illness in schizophrenia [76]. Other diseases such as attention deficit hyperactivity disorder (ADHD) and Alzheimer’s disease (AD) can also affect the properties of fMRI brain networks according to several recent studies. For example, previous studies suggested that global efficiency decreased and local efficiency increased in the brain networks of ADHD subjects at a wide range of cost thresholds (a wide range of cost thresholds was specifically employed to investigate network efficiency) [77]. Additionally, patients with AD showed increased connectedness and randomization in the small-world model [78]. In the analysis of weighted networks derived from resting-state MEG data, the study showed a decreased clustering coefficient and increased path length in the patients with AD [79]. Individual variables cannot be ignored. The individual variables in the actual measures include physiological noise [80], in-scanner head motion [81], the condition of the resting state [82], scan length [83], quality of registration [84], etc. Yan et al. have shown that head motion in fMRI can bring about increasing local efficiency while decreasing global efficiency and small worldness [55]. In the analysis of brain networks, head motion should be corrected. Although many of the methods discussed above were used to investigate brain networks, quantifying brain connectivity efficiently and accurately still remains a challenging problem [85]. Therefore, the impacts of diseases should be reflected in improved atlases.

## Further considerations

Whether the degree of node distribution in brain networks follows an exponentially truncated power law or a scale-free degree distribution (power law) is still disputed [24]. However, the dispute has not obstructed the verification of the atlas-based impact on brain networks. Studies of brain networks based on different atlases on different scales and on the same scales both showed that atlases affected the quantification of network properties (small-world and scale-free properties). This impact on quantification was a comprehensive result, combining the impacts of atlases and evaluation algorithm of network properties. In the estimation of small-world properties, the construction of random networks was very critical. Random networks should have the capacity to reduce the impacts of methods to construct a connectivity matrix on small-world properties and provide network properties close to true random networks. As Zalesky et al. described in their studies, appropriate null networks should be used to benchmark network measures in correlation networks [86]. Otherwise, the extent of small-world properties might be overestimated with full correlation and underestimated with partial correlation [86]. Besides quantification of network properties, quantification of the brain network was also affected. Most analyses of structural and functional networks were based on undirected and unweighted graphs, which might be related to the accuracy of quantification of the brain network. Poor quantification could cause an accumulation of the impacts and lead to results far from those expected.

Most of the atlases used were derived from anatomical landmarks or cytoarchitectonic boundaries. These atlases contained little information about connectivity, so their capacity for accurately representing connectomes was limited [21]. Using clustering theory, atlases could be improved based on connectivity, such as the CC200/CC400 proposed by Craddock et al. [74]. In their papers, they summarize some criteria for evaluating the suitability of a set of regions of interest for whole-brain resting-state functional connectivity analyses. Of course, many other factors should be taken into account to improve the atlases, such as most of the cortex being buried in the sulcal folds [87], gene expression and dynamic functional connectivity. Optimized atlases can be developed by meta-analyses, such as the Dosenbach 160-region and Power 264-region atlases.

The constructed brain networks also can be grouped into two classes: single-subject and group networks. Probabilistic atlases might be not suitable for construction of single networks. In the analysis of functional networks, both single and group networks were sometimes used, so an atlas balancing interindividual and inter-group differences might be needed. In the practical use of atlases, the segment of individual neuroimaging and a common coordinate space affect the use effect. When building atlases, how to optimally use them should be taken into account. Multi-atlas segments might be a method to make better use of them; this could generate more accurate structural segmentations of the brain by combining prior anatomical information from multiple atlases [88, 89]. A multi-atlas could also reduce the negative impacts from registration errors [90]. As a result, a multi-atlas might be an important approach to matching atlas information and the subjects used.

## Conclusion

Although atlases have a lesser effect in the determination of brain network properties, they have a great influence on the quantification of brain network properties. Improved atlases can facilitate the quantification of brain networks and the introduction of more network theories. The selection of atlases is important, and cortex parcellation should be improved based on the function and structure of the brain. Parcellation should also balance inter-individual and intergroup differences. Additionally, the new theories need to be applied in studies related to unknown fields in the brain network. With the development of the technology, a stronger magnetic field might be employed and higher resolution images acquired, which would greatly promote studies in brain science.
